# Evolutionary dynamics of chromosomal races in the Japanese allotetraploid spined loach, *Cobitis* sp. ‘yamato’ species complex

**DOI:** 10.1371/journal.pone.0351665

**Published:** 2026-11-11

**Authors:** Hayato Oka, Yuki Yashima, Takeru Uechi, Takafumi Fujimoto, Tadao Kitagawa

**Affiliations:** 1 Graduate School of Agriculture, Kindai University, Nara, Japan; 2 Graduate School of Fisheries Sciences, Hokkaido University, Hakodate, Hokkaido, Japan; National Cheng Kung University, TAIWAN

## Abstract

The Japanese spined loach, *Cobitis* sp. ‘yamato’ species complex (hereafter *Yamato* spined loach), is an allotetraploid group originating from two diploid *Cobitis* species hybridization*.* Although specific regional populations have ancestral 2n = 98, decreases in 2n have been reported, indicating ongoing rediploidization. However, distribution patterns and phylogenetic relationships of each chromosomal race remain unknown. Using conventional cytogenetics, we identified seven 2n values across 45 populations (2n = 80, 82, 86, 90, 92, 94, and 98), including two newly reported karyotypes (2n = 80 and 92). Five races clustered in specific geographical areas, whereas two races (2n = 80 and 90) formed discontinuous distribution ranges. Mitochondrial phylogenies (*cytb* + *ND2*) revealed three major clusters with shallow differentiation and ancestral polymorphism retention across races, indicating rapid rediploidization. Races with few chromosomes belonged to derivative clusters, which reflect a gradual chromosome number reduction during *Yamato* spined loach evolution. Polyphyly within races indicates parallel and independent chromosomal reductions. Flow cytometry showed no positive correlation between chromosome number and relative DNA content, suggesting that karyotype rearrangements, rather than the loss of specific chromosomes, contributed to the decrease in chromosome or fundamental numbers.

## Introduction

Modern vertebrates have experienced several whole-genome duplications (WGD) [1]. Such polyploidization events are followed by rediploidization via deletion of the duplicated genome, playing a crucial role in vertebrate evolution [2,3]. As most modern vertebrates are paleopolyploid species that have undergone rediploidization [3–5], understanding the rediploidization process is difficult. Genomes downsize after polyploidization, indicating duplicated homoeologous gene loss [6,7]. Although extensively studied in plants [8], rediploidization involving a decrease in chromosome number [3] remains insufficiently understood in vertebrate lineages. To understand vertebrate genome evolution, studying organisms that have recently experienced WGD while undergoing chromosome number reduction would be ideal.

Loaches of the genus *Cobitis* (family Cobitidae) are freshwater fishes distributed in temperate Eurasia and its surrounding regions [9]. Twenty species/subspecies inhabit Japan [10], most of which are diploid species. However, several auto- and allo-tetraploid species exist [10–13]. The taxonomy of Japanese *Cobitis* is complex and remains unsettled, with several lineages being referred to by informal or provisional designations [10]. Throughout this manuscript, such informal or provisional taxonomic designations are cited with “sensu” followed by the author(s) and year of the study in which the name was used in that sense (e.g., *C.* sp. BIWAE type A sensu Nakajima et al. 2012). Japanese diploid species of *Cobitis* have 46–50 chromosomes (modal number: 50), whereas Japanese tetraploid species have 82–98 chromosomes [13,14]. Tetraploid individuals of this genus have also been reported in Europe [15]. Polyploid loaches are likely derived from diploid asexual hybrid populations via a triploid bridge; however, most tetraploid individuals are inviable, or males are sterile [15,16] In contrast, all Japanese tetraploid species reproduce bisexually and have escaped an unstable asexual reproductive mode (*e.g., Cobitis magnostriata* Nakajima 2012, *C. takenoi* Nakajima 2016, and *C.* sp. BIWAE type A sensu Nakajima et al. 2012) [11].

*Cobitis* sp. ‘yamato’ species complex sensu Nakajima et al. 2012 inhabits the western tip of Honshu and Kyushu Islands and is of allotetraploid origin, resulting from the hybridization between the diploid species [17]. The hypothetical original number of chromosomes in this species complex was estimated at 98 based on a combination of the parental species of the *Cobitis biwae* species complex and *Cobitis striata* species complex, with 48 and 50 chromosomes, respectively [13]. The *Cobitis* sp. ‘yamato’ species complex was formerly described as *Cobitis matsubarae* Okada & Ikeda, 1939, and a part of the population inhabiting Oyodo River in southern Kyushu was described as an independent species, *Cobitis sakahoko* Nakajima & Suzawa 2016. However, the taxonomic status of this species complex remains unresolved due to the possible inclusion of some cryptic species [17–19]. Hereafter, we refer to this species complex as *Yamato* spined loach after its common Japanese name (*Yamato–shima–dojo*).

The *Yamato* spined loach species includes geographically clustered populations with various unique chromosome numbers (range: 82–98) [13,17,19,20]. However, chromosomal population distribution patterns are limited to certain areas of their habitat, specifically northern Kyushu and part of Honshu. Therefore, the specific distribution of each chromosomal population in the range remains unknown. Although a specific regional group of *Yamato* spined loach has 98 chromosomes, others have fewer chromosomes, indicating that the loach is undergoing a rediploidization process. Because differences in karyotypes are closely linked to biological species boundaries [21,22], each population with a different number of chromosomes likely establishes post-mating isolation from other chromosomal populations. As mentioned above, the taxonomic status of *Yamato* spined loach is unresolved; therefore, we tentatively consider population groups with different chromosome numbers as independent chromosome ‘races.’ *Cobitis sakahoko,* whose chromosome number remains unknown, is genetically and morphologically distinguishable from other *Yamato* spined loach [23]. We therefore refer to it as the *Oyodo* race, following its Japanese common name (*Oyodo-shima-dojo*), irrespective of its chromosome number.

The *Yamato* spined loach experienced allotetraploidization relatively recently (approximately 3.6 million years ago [MYA]) compared with that in other polyploid vertebrates (for example, *Xenopus laevis*, approximately 18 MYA [6]; *Cyprinus carpio*, approximately 12 MYA [7]) and is a unique vertebrate showing a continuous chromosome number reduction process from allotetraploid to diploid. Therefore, this loach might be an ideal organism for understanding rediploidization after WGD. Understanding the chromosome number reduction mechanism in loaches and relationships among races could provide valuable insights into WGD evolution and vertebrate diversification.

In this study, we determined the chromosome numbers of each population to identify the distribution of each different chromosome race and performed mitochondrial DNA (mtDNA) phylogenetic analysis to reveal the genetic relationships among the races.

## Materials and methods

### Specimens

Individuals of the *Yamato* spined loach with unknown chromosome numbers were collected from 45 populations across 36 river systems. Locations were selected from 55 river systems covering the entire distribution range of the *Yamato* spined loach, as reported in previous studies [10,18,23–27] (Fig 1; S1 Table). The populations of the *Yamato* spined loach were not large, and at least three individuals from each population could be collected (S2 Table). Fish collection did not require any site-specific permits, as the *Yamato* spined loach is not designated as a protected species under Japanese law, and no fishing rights under the Inland Water Fisheries Coordination Regulations had been established for any of the sampling rivers. All collections were conducted using dip/seine nets with mesh sizes permitted under local fishing gear regulations, and fish were handled in accordance with the guidelines for the use of fish as research material established by the Ichthyological Society of Japan (2003; revised 2024; https://www.fish-isj.jp/html/iin/nature/guideline/2003.html).

**Fig 1:**
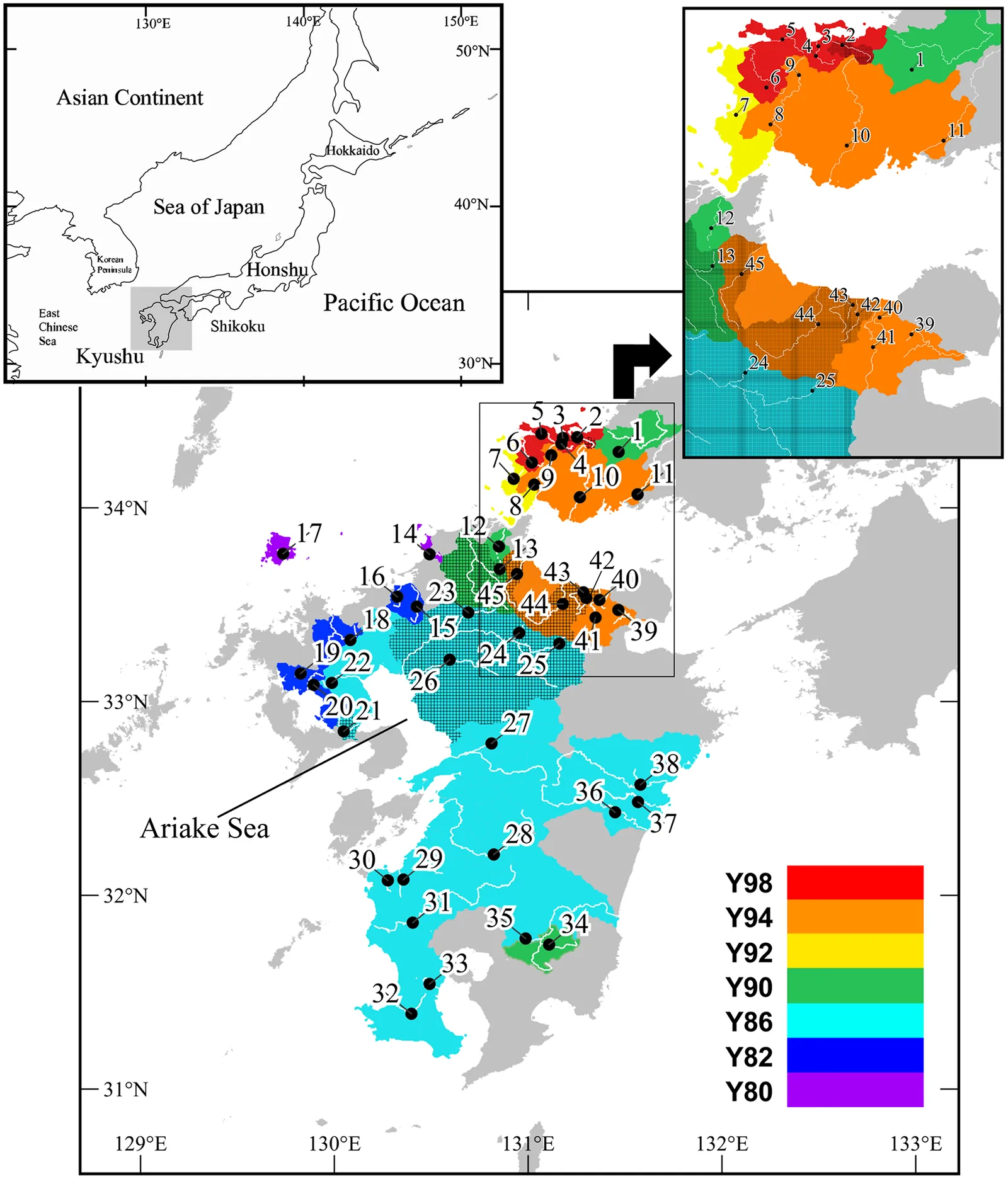
Sampling locations of the *Yamato* spined loach (*Cobitis* sp. ‘yamato’ species complex) with location codes and distribution patterns of each of the chromosomal races of *Yamato* spined loach in Japan. The cross filled area is the known distribution patterns of chromosomal races of them. The numbers correspond to those in S1 Table. Administrative boundaries are based on the ‘National Land Numerical Information (Administrative Areas)’ (Ministry of Land, Infrastructure, Transport and Tourism of Japan; https://nlftp.mlit.go.jp/ksj/gml/datalist/KsjTmplt-N03-2026.html), used under a CC BY 4.0 license. River data are derived from OpenStreetMap contributors (https://www.openstreetmap.org/copyright), available under the Open Database License (ODbL).

### Chromosome preparation

Chromosome preparation was performed as previously reported [20], with slight modifications. The loaches were anesthetized using 2-phenoxyethanol, and the kidney tissues were removed and minced with scissors. Obtained cells were incubated for 2 h at 25 °C in Eagle’s minimal essential medium (Nissui, Tokyo, Japan) containing colchicine (0.10–0.15 μg/mL). Following KCl (0.067 M) hypotonization for 20 min, the solution was replaced three times with a methyl alcohol:acetic acid fixative (3:1) and centrifuged at 150 g for five minutes. Cell suspension drops were air-dried and stained with Giemsa. Chromosomal images were observed under a microscope at 1000 × magnification and captured using Visualix STD1 (Visualix Corporation, Hyogo, Japan) to count the number of chromosomes. At least 10 metaphase cells per specimen were analyzed, and the chromosome numbers from individual specimens did not vary in most cases. Karyotypes were determined only in populations with more than 10 complete metaphases and proper spreading. Considering the difficulty in determining the karyotype for the entire population because of the high number of chromosomes, the karyotypes of known chromosomal races were also determined by comparison with those described in previous studies [13,19]. The chromosome nomenclature was determined according to Levan et al. [28]. Neither arm ratio nor chromosome size consistently showed specific values owing to the degree of arm contraction caused by colchicine treatment. Therefore, arm ratios were determined on an average basis using several nuclei. The fundamental number (FN) was calculated by counting acrocentric chromosomes as having a single arm and all other chromosomes as having two arms, following karyotype analysis of *Yamato* spined loach [13].

### Flow cytometry

To examine whether the decrease in chromosome number was associated with subsequent DNA loss in each race of *Yamato* spined loach, relative DNA content was measured using flow cytometry. The fin tissues cut from each specimen were processed using a standard protocol for nuclear staining with 4′,6-diamidino-2-phenylindole (DAPI), and the relative DNA content was determined using a Partec CyFlow Ploidy Analyzer (Partec GmbH, Münster, Germany). A closely related diploid species, *Cobitis* sp. BIWAE type B sensu Nakajima et al. 2012 (2n = 48) was used as a control. Fifty-nine individuals from eight different races, including at least one population representing all chromosomal races, were analyzed and compared (S2 Table). Correlations (chromosome number versus DNA content and FN versus DNA content) were examined using the obtained data.

### DNA analysis

The right pectoral fins of individuals used for karyotyping were preserved in 99.5% ethanol for DNA analysis. The rest of the body was fixed in 10% formalin and preserved in 70% ethanol. The specimens were deposited at the Kindai University Nara Collection (KUN–P65265 to 66698; not all numbers applied). To elucidate the phylogenetic relationships among the races, phylogenetic trees were constructed based on *cytochrome b* (*cytb*) (1,051 bp) and *NADH dehydrogenase 2* (*ND2*) (1,025 bp) mtDNA. Total DNA was extracted from ethanol-preserved fin tissues as previously described [29]. Polymerase chain reaction (PCR) was performed to amplify the *cytb* region using Glu-L. Ca14337–14359 (5′-GAA GAA CCA CCG TTG TTA TTC AA-3′) and Thr-H. Ca15598–15548 (5′-ACC TCC RAT CTY CGG ATT ACA-3′) primer pairs [30]. The PCR conditions were as follows: 95 °C for 2 min; 30 cycles of 95 °C for 30 s, 55 °C for 30 s and 72 °C for 1 min; and 72 °C for 5 min. *ND2* PCR was performed using two newly designed primers, MT3982_ND2F (5′-TAA GCT TTC GGG CCC ATA CC-3′) and MT5180_ND2R (5′-AGT GTC TGG CTT GCA TTC AGA-3′), according to mtDNA sequences of *C. matsubarae* (NC_029441). The PCR conditions were the same as those for *cytb* except the annealing temperature, which was set at 56°C. PCR amplification was performed in a 20 µL reaction volume containing 2 µL of template DNA, 1 × reaction buffer, 4 mM MgCl_2_, 0.2 mM each dNTP, 1 µM each of forward and reverse primers, and 0.5 U of BIOTAQ DNA polymerase (Meridian Bioscience/Bioline). The PCR products were subjected to polyethylene glycol precipitation [31], with slight modifications. Sequencing was performed in both directions using the same set of primers described above. Sequencing data were obtained from the Macrogen Japan Corporation sequencing facility, equipped with a 3730xl DNA Analyzer (Applied Biosystems, Waltham, Massachusetts, USA), and submitted to the DDBJ/EMBL/GenBank databases under accession numbers LC819018–LC819106 (S3 Table).

### Phylogenetic analysis

Multiple alignments of nucleotide sequences were performed using the ClustalW option in MEGA 11 ver. 0.13 [32], and the alignment was manually corrected. Two mitochondrial genes, *cytb* and *ND2*, were considered as a single locus and analyzed. Phylogenetic trees were constructed using neighbor-joining (NJ) and maximum-likelihood (ML) methods. NJ analysis was performed using MEGA11. A distance matrix was calculated using Kimura’s two-parameter method [33] following a previous phylogenetic study [34]. ML analysis was performed using RaxML ver. 8.2.10 [35] with partitions according to three codon positions. The appropriate substitution model was determined using the Kakusan4 ver. 4.0 [36]. A substitution general time-reversible (GTR) model with gamma radiation was applied to each partition. Two sets of bootstrap values (1,000 replications for NJ and ML analyses) were calculated to obtain node support measures for the resulting tree [37]. *Cobitis* sp. BIWAE type C sensu Nakajima *et al.* 2012, *C. striata striata*, *Niwaella delicata*, and *Misgrnus dabryanus* were included as outgroups based on previously known mtDNA phylogenetic relationships [30,34,38]. To assess the probability of support for the monophyly of each race, the RaxML ver. 8.2.10 [35] was used to compute the log-likelihood values of the constrained topology with the GTR using a gamma substitution model. The SH [39] and AU tests [40] were then performed using CONSEL ver. 0.20 [41] to calculate *p*-values of the topologies for comparison.

### Haplotype network

To visualize the detailed relationships among each chromosomal race in *Yamato* spined loach, genealogical relationships between the concatenated *cytb* and *ND2* haplotypes were constructed using the TCS algorithm [42] in PopART ver. 1.7 [43], except for the haplotypes distant from the major haplotypes (H55 and H56). Ambiguous (multiple) loops in the networks were resolved using criteria based on the coalescent theory [44], as previously summarized [45].

### Ethical considerations

The survey and all the experiments complied with Japan’s current laws. The *Oyodo* race (*Cobitis sakahoko*) considered in this study is listed as endangered on the IUCN Red List. To minimize the impact on the population, only three individuals were collected from their habitat, and the collected fish were euthanized using 2-phenoxyethanol before body fixation in 10% formalin to minimize suffering and meet acceptable ethical standards.

## Results

### Chromosome numbers

Chromosome numbers were determined for individuals collected from 40 populations across 35 river systems and the detailed distribution pattern of each chromosomal race of *Yamato* spined loach was determined using a combination of known data (S1 Table; Fig 1). Seven 2n chromosome numbers, including two novel numbers (2n = 92 and 80), were obtained from *Yamato* spined loach and are hereafter referred to as the Y98, Y94, Y92, Y90, Y86, Y82, and Y80 races.

Individuals belonging to a particular chromosomal race were geographically restricted to specific areas (S1 Table; Fig 1). The Y98 race was obtained from the Fukawa and Misumi Rivers on the Sea of Japan side of western Honshu. The Y94 race was detected in rivers on the Seto Inland Seaside of western Honshu and northeastern Kyushu. The Y92 race was collected only from the western tip of Honshu. The Y90 race was obtained from two discontinuous areas, the Sea of Japan side of western Honshu (Abu-R.) and northern Kyushu (Onga- and Murasaki-R.), referred to as Y90a and Y90b, respectively. The chromosome number of the *Oyodo* race, which inhabits only Oyodo-R. in southeastern Kyushu, was also 90. The Y86 race originated from river systems flowing into the Ariake Sea and those in southern Kyushu. The Y82 race was collected from rivers flowing into the Sea of Japan in Kyushu. The Y80 strain was obtained from Hanhoko-R. on Iki Island (Y80a), and Saigo-R. flowed into the Sea of Japan at Kyushu (Y80b).

The 98 chromosomes constitution of Fukawa- and Awano-R. populations contained 11 metacentric, 14 submetacentric or subtelocentric, and 24 acrocentric pairs (FN = 148) (Fig 2a). The karyotype of the newly obtained Y94 race could not be determined because chromosome spreads of sufficient quality for karyotype analysis were not obtained. The 92 chromosomal constituents included 12 pairs of metacentric chromosomes, 14 pairs of submetacentric or subtelocentric chromosomes, and 20 pairs of acrocentric chromosomes (FN = 144) (Fig 2b). The 82 chromosomes in Ishiki-R. constituted 20 metacentric pairs, 13 submetacentric or subtelocentric pairs, and eight acrocentric pairs (FN = 148) (Fig 3b). In the Y90 race, two different karyotypes were observed: the Y90a race with 17 metacentric, 10 submetacentric or subtelocentric, and 18 acrocentric pairs (FN = 144) (Fig 2c), and the Y90b race with 16 metacentric, 13 submetacentric or subtelocentric, and 16 acrocentric pairs (FN = 148) (Fig 2d). The chromosomal constitution of *Oyodo* race, with 90 chromosomes, also exhibited different karyotypes: 11 pairs of metacentric chromosomes, 17 pairs of submetacentric or subtelocentric chromosomes, and 17 pairs of acrocentric chromosomes (FN = 146) (Fig 3a). The Y80a and Y80b races also showed two different karyotypes: the Y80a race had 24 metacentric, 13 submetacentric or subtelocentric, and three acrocentric pairs (FN = 154) (Fig 3c); the Y80b race had 25 metacentric, 11 submetacentric or subtelocentric, and four acrocentric pairs (FN = 152) (Fig 3d).

**Fig 2:**
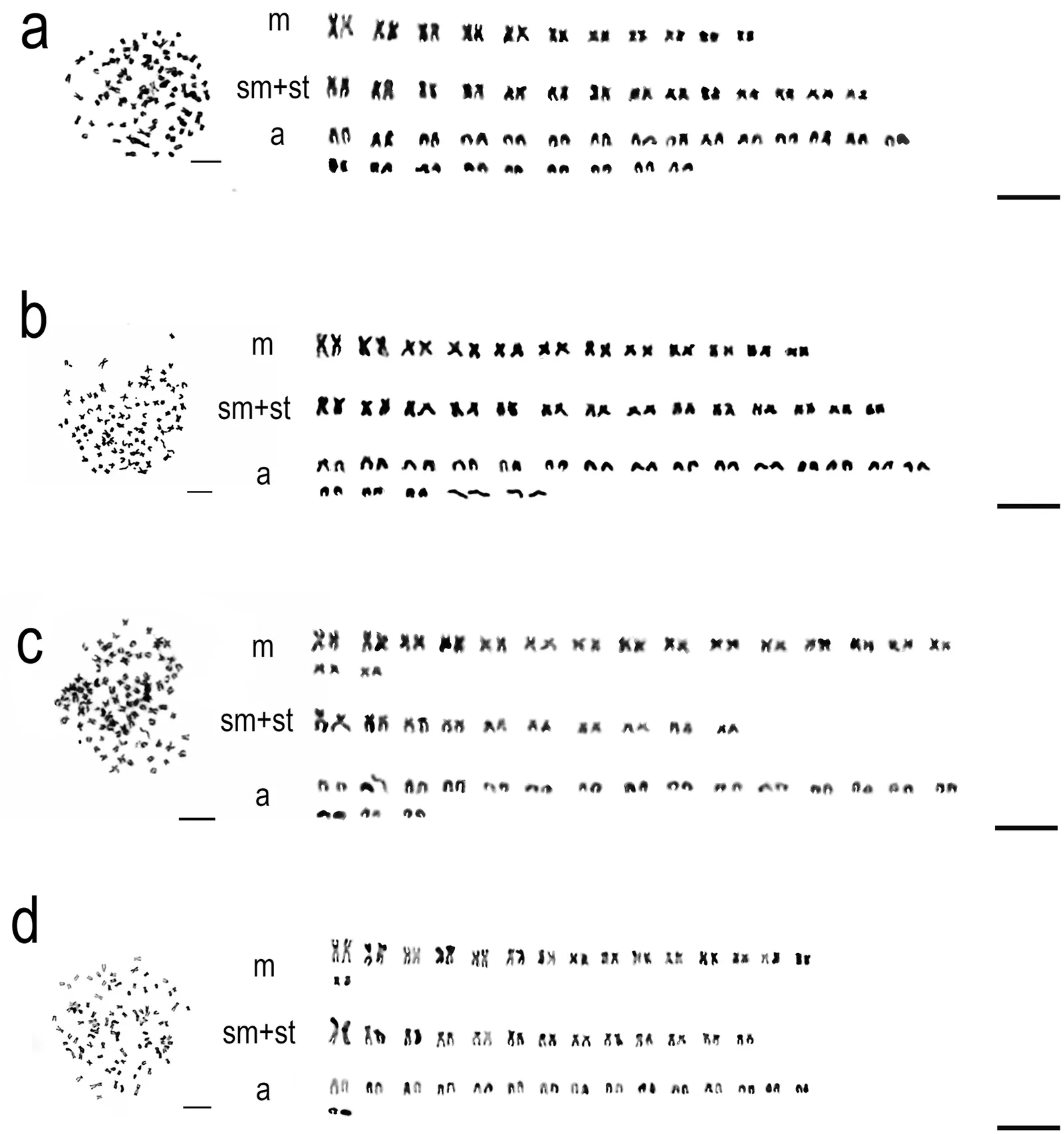
Karyotype of each race of the *Yamato* spined loach. (a) 2n = 98: 11 metacentric pairs (M) + 14 submetacentric or subtelocentric pairs (SM, ST) + 24 acrocentric pairs (A), from the Awano River. (b) 2n = 92: 12M + 14SM, ST + 20A, from the Kawatana River. (c) 2n = 90 (Y90a race): 17M + 10SM, ST + 18A, from the Abu River. (d) 2n = 90 (Y90b race): 16M + 13SM, ST + 16A, from the Murasaki River.

**Fig 3:**
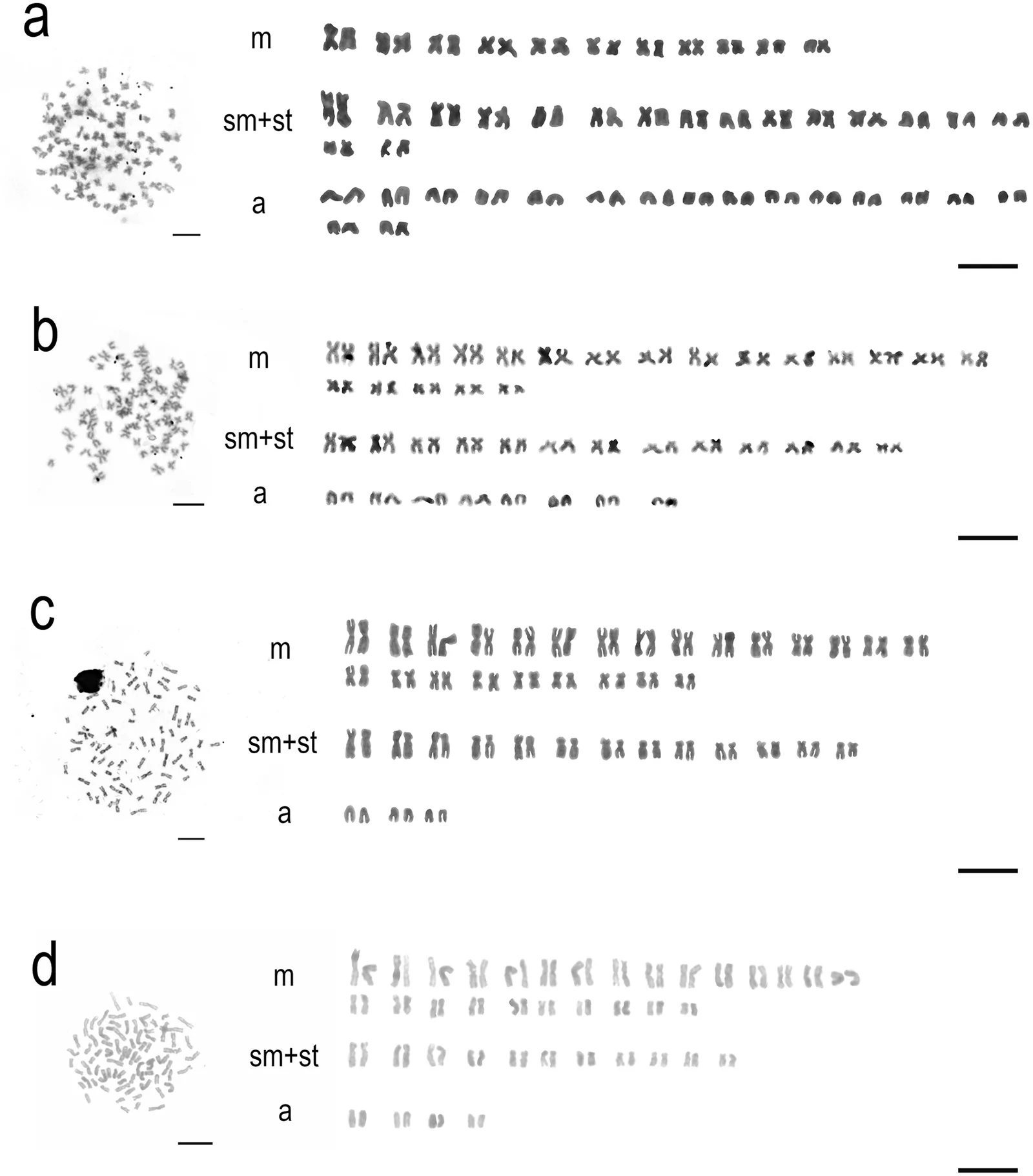
Karyotype of each race of the *Yamato* spined loach. (a) 2n = 90 (*Oyodo* race): 11M + 17SM, ST + 17A, from the Oyodo River. (b) 2n = 82: 20M + 13SM, ST + 8A, from the Kawatana River. (c) 2n = 80 (Y80a race): 24M + 16SM, ST + 3A, from the Saigo River. (d) 2n = 80 (Y80b race): 25M + 11SM, ST + 4A, from the Hanhoko River. Scale bars indicate 10 μm.

### Flow cytometric analysis

The range of relative DNA content compared with the content of the diploid control (set as 1.000) was 1.914–2.212 in *Yamato* spined loach (Fig 4). Y94 (Yakkan R) and Y82 had the lowest and highest DNA content among the analyzed populations, respectively. Chromosome number or FN and relative DNA content was not correlated (correlation coefficient = −0.62 and −0.48; r^2^ = 0.39 and 0.23).

**Fig 4:**
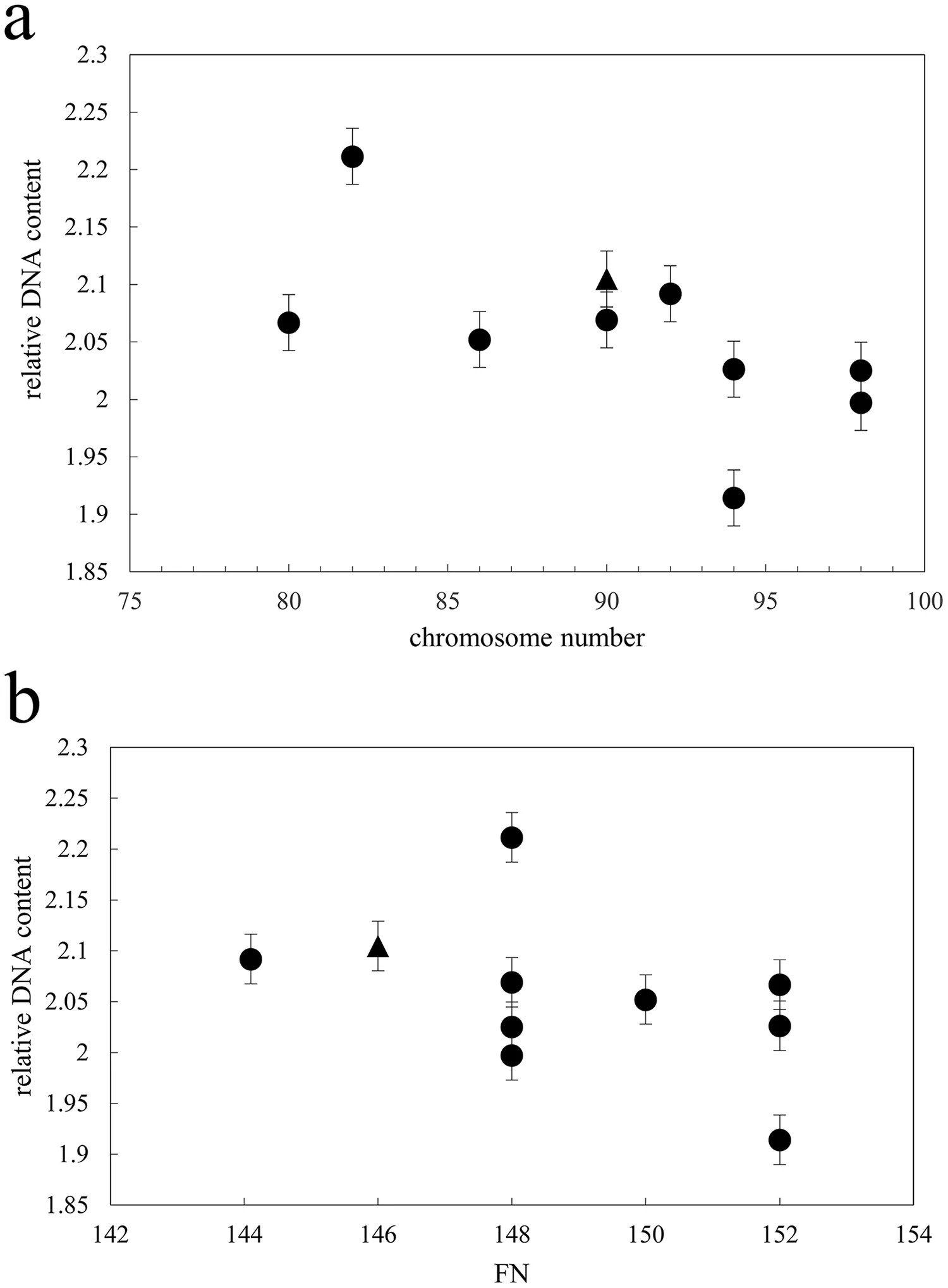
Scatter plot of (a) chromosome numbers and (b) arm number of chromosomes (FN), against the relative DNA content of the *Yamato* spined loach. A triangle plot shows the *Oyodo* race (see the text).

### DNA analysis

In the phylogenetic trees based on the *cytb* + *ND2* region, haplotype Type A (known as a polymorphic mtDNA haplotype within a common reproductive population included only in specific populations (Fukawa-R.) [18]) differed significantly from other haplotypes from *Yamato* spined loach (Type B). The close relationship between *Cobitis* sp. BIWAE type D sensu Nakajima et al. 2012, and *Cobitis magnostriata* of haplotype type A corresponded to those reported in a previous study [18]. All individuals from Fukawa-R. had 98 chromosomes, irrespective of the mtDNA type (S1 Table). The *Oyodo* race (with 90 chromosomes) branched from the relatively highly supported cluster of other common haplotypes of *Yamato* spined loach, the Type B haplotype [18]. Although the branches among the Type B haplotypes generally showed shallow differentiation, four subclusters, three relatively highly supported (subclusters B1–B3) and one poorly supported (B4), were formed (Fig 5).

**Fig 5:**
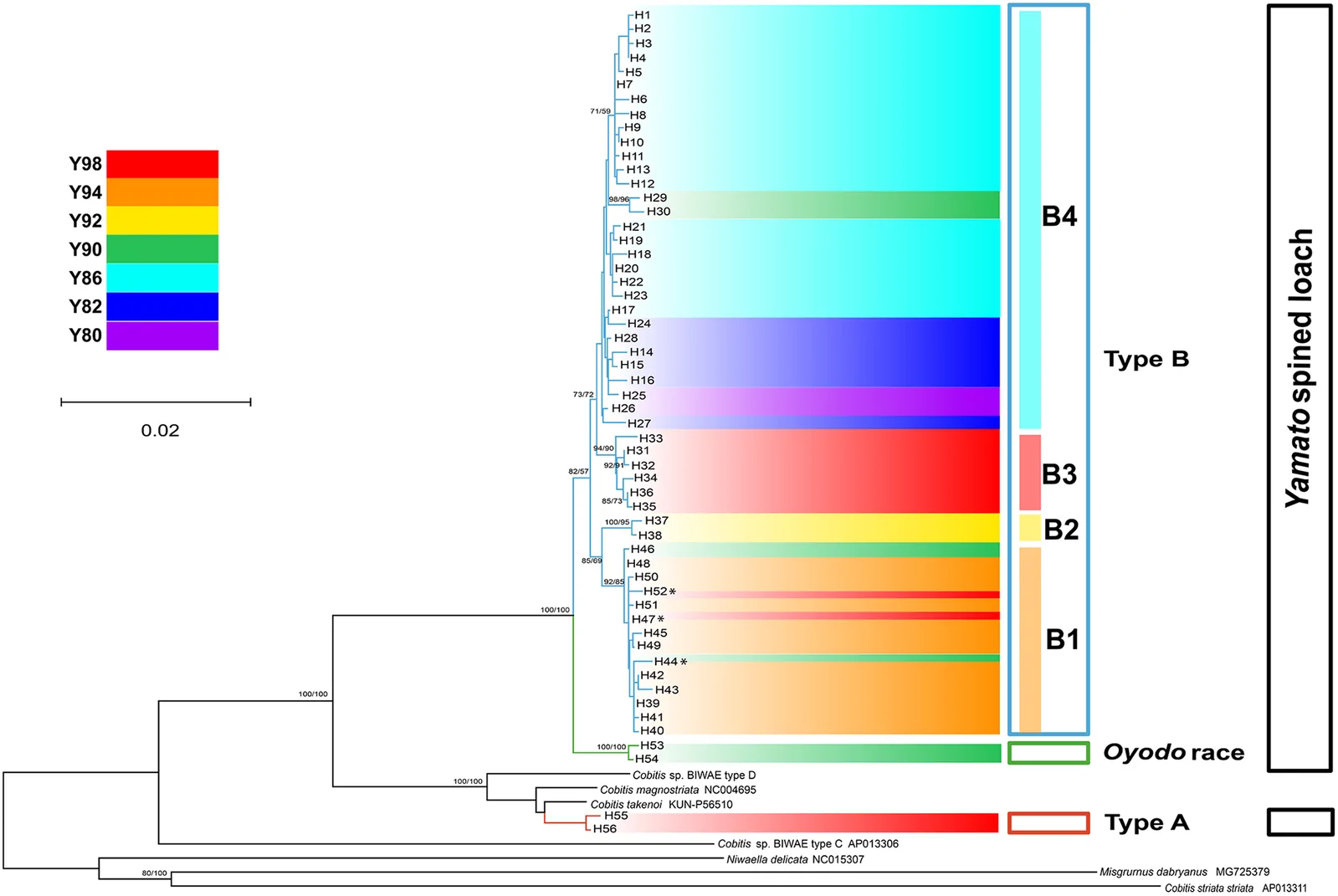
Neighbor-joining (NJ) tree based on ND2 sequences (1,025 bp) + cyt*b* sequences (1,051 bp) from the *Yamato* spined loach and the related species with bootstrap probabilities (NJ and ML, > 70%) of 1,000 resampling. The tree is rooted with *Cobitis striata striata*, *Misgurnus dabryanus*, *Niwaella delicata*, and *Cobitis* sp. BIWAE type C as outgroups. Subclades (Type A & B and *Oyodo* race) names are given. Haplotypes (H1–H56) correspond to those in S1 Table. Filled red, orange, green light blue, pink and purple symbols indicate the chromosomal races. The asterisks represent haplotypes with two different chromosomal races.

After mapping each chromosome number onto the phylogenetic tree, high numbers of chromosomes (Y98, Y94, and Y92) belonged to three well-supported sub-clusters (B1–B3) and tended to be included in the respective independent sub-clusters (Y98 in B3, Y94 in B1, and Y92 in B2). Few chromosomes (Y86, Y82, and Y80) were included in subcluster B4 (Fig 5). Common haplotypes of Y94 race (H47 and H52) were detected in the Y98 population. Two geographically separated karyotype races with 2n = 90 (Y90a and Y90b) were included in different subclusters. Monophyly of the Y98 and Y90 races was rejected in both the AU and SH tests (Table 1).

**Table 1:** Topology test for the eight constrained topologies of each race in the *Yamato* spined loach.

Constraints of monophyly	Topology	AU test *p*-values	SH test *p*-values
Y98 race (except type A)	(H31, H32, H33, H34, H35, H36, H47, H52)	2e-005*	0.001*
Y98 race	(H31, H32, H33, H34, H35, H36, H47, H52, H55, H56)	0.001*	0*
Y94 race	(H39, H40, H41, H42, H43, H44, H45, H47, H48, H49, H50, H51, H52)	0.342	0.348
Y92 rae	(H37, H38)	0.423	0.415
Y90 race	(H29, H30, H44, H46)	3e-006*	0.001*
Y90 race+*Oyodo* race	(H29, H30, H44, H46, H53, H54)	3e-009*	5e-004*
Y86 race	(H1, H2, H3, H4, H5, H6, H7, H8, H9, H10, H11, H12, H13, H17, H18, H19, H20, H21, H22, H23)	0.455	0.504
Y82 race	(H14, H15, H16, H24, H27, H28)	0.199	0.286
Y80 race	(H25, H26)	0.019*	0.077

*p-value <0.05 indicates statistical rejection.

### Haplotype network analysis

Besides H55 and H56 (Type A), the haplotype networks showed differentiation between the *Oyodo* and Type B haplotypes, as well as between certain cohesions of each chromosomal race (Fig 6). Although haplotypes with higher chromosome number races tended to form independent clusters in the networks, those with lower chromosome number races tended to continuously form a single cluster. Two karyotype races with 90 chromosomes (Y90a and Y90b) were detected at different positions in the network.

**Fig 6:**
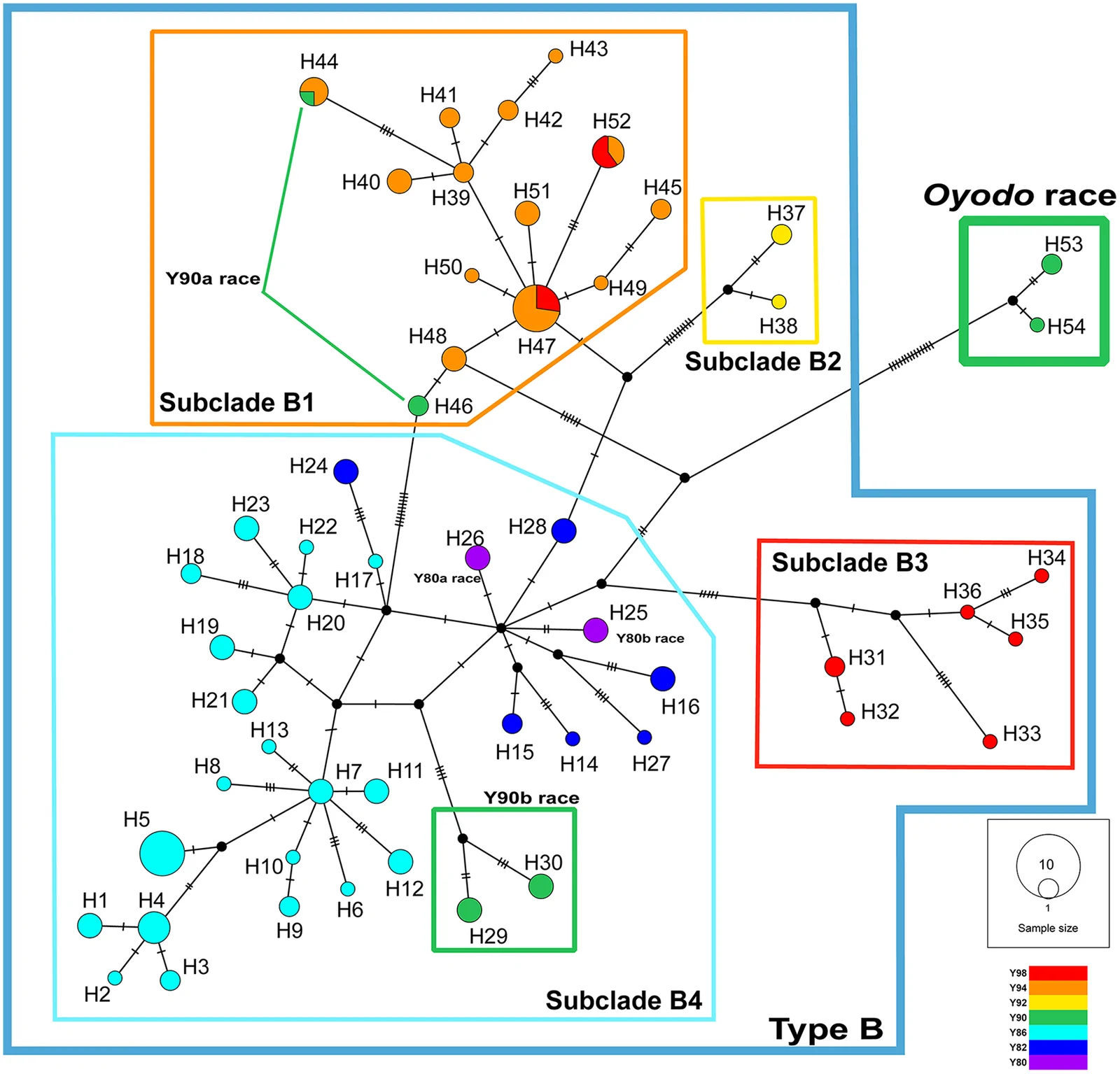
Statistical parsimony networks of the *Yamato* spined loach based on ND2 sequences (1,025 bp) + cyt*b* sequences (1,051 bp). The circle size reflects the number of specimens of a particular haplotype. The number near each circle indicates haplotype shown in S1 Table. Small circles without numbers signify possible missing haplotypes. Haplotype colors match each chromosomal race.

## Discussion

The existence of the Y80 race, which has been reduced by 18 chromosomes from the putative ancestral Y98 race, indicated a more advanced evolutionary process than previously thought. The highest sequence divergence among the common Type B, based on Kimura’s two-parameter method [33], was observed between the race with the highest chromosome number (Y98; H52) and that with the lowest (Y80; H25), which corresponds to an estimated divergence time of approximately 0.37 MYA, under an assumption of the molecular clock rate of the cyprinid *cytb* (0.76% per million years; [46]). Shallow genetic differentiation and retention of common haplotypes among chromosomal races of *Yamato* spined loach indicated rapid rediploidization within the *Yamato* spined loach. The allotetraploidization event in the *Yamato* spined loach is estimated to have occurred after approximately 3.6 MYA, based on the divergence time between the *Yamato* spined loach complex and its closest maternal species, *C.* sp. BIWAE type D. This polyploidization event in the *Yamato* spined loach is more recent than that of other reported polyploid (or paleopolyploid) species, such as *X. laevis* (17–18 MYA; [6]) and *Cyprinus carpio* (12 MYA; [7]).

Although high-chromosome number races tended to form differentiated clusters in the phylogenetic tree, the low-chromosome number races tended to belong to a single derivative cluster, suggesting a stepwise and gradual reduction in chromosome number during evolution. The polyphyly of races with 90 chromosomes (Y90a, Y90b, and *Oyodo*) indicated that part of the reduction in chromosome number was parallel and independent in *Yamato* spined loach.

High-chromosomes (≥90) races, inferred as older-lineage chromosomes, were obtained from northern Kyushu, the western tip of Honshu, and the southeastern part of Kyushu (Fig 1). The continuous distribution pattern of the Y94 race across the two islands can be explained by the connection of rivers through a large riverine system during the last ice age that flowed into the riverine systems of the Seto Inland Sea and Suo-nada [47]. Conversely, populations bearing different chromosome numbers largely correspond to river systems that lack such connections, either historically or at present. This association suggests that geographical isolation has allowed chromosomal reorganization to proceed independently within each system. The distributions of Y90a and Y90b also ranged from the areas adjacent to the Y94 race. The distribution of high (Y90 and more) chromosome races can be, therefore, regarded as a continuous area located in the eastern part for *Yamato* spined loach, except for that of *Oyodo* race. In contrast, the Y86 race, which is continuously distributed over a wide range in Kyushu, showed a uniform karyotype (13 metacentric, 16 submetacentric or subtelocentric, and 11 acrocentric pairs [FN = 150]) and low genetic diversity, indicating that the Y86 race has rapidly spread across its current range. In Kyushu, two closely related native bagrid catfish species, *Tachysurus* (Siluriformes: Bagridae), are allopatrically distributed. Similarly, the distribution pattern of races with a high number of chromosomes (≥90) corresponded to that of *Tachysurus nudiceps*, whereas the pattern of a low number of chromosomes (≤86) corresponded to that of *T. aurantiacus* [24,48], suggesting common geographical differentiation events or features between the areas.

The lower chromosome number races (Y82 and Y80) were suggested to be in their initial endemic status, owing to their limited distribution and derivative phylogenetic position from Y86. Reciprocal monophyletic relationships between the two Y80 haplotypes detected in distantly located populations with different karyotypes were sufficient to reject one of the statistical tests (Table 1), which also suggested an independent reduction of the chromosomes in each location.

In allotetraploid species, the older populations are estimated to contain the highest number of original chromosomes [3]. An exceptional case based on these rules was partially recognized. Haplotypes (H47 and 52) from the Y98 race in Misumi and Fukawa-R. were similar to those of the Y94 race, forming an independent subcluster (B1). Although the possibility that the population increased the chromosome number from 94 to 98 cannot be completely excluded, the population originally had 98 chromosomes, and hybridization with the Y94 race from river capture might have brought mtDNA introgression to the Y98 race.

In Teleostei, rediploidization processes have been reported in several auto- and allopolyploid fishes based on genome-wide ohnolog divergence (e.g., salmonid fishes [49] and cyprinid fishes [7,50]). However, to our knowledge, no previous study has documented rediploidization processes spanning multiple sequential stages after polyploidization within a single allopolyploid lineage, as observed in the present study. The present flow cytometric analysis indicated that the DNA content of each race was different; however, positive correlations between either chromosome number or FN and DNA content were not observed. This result suggests that karyotype rearrangements (mainly Robertson translocation or tandem fusion), rather than the loss of specific chromosomes, may have contributed to the decrease in chromosome number. The *Yamato* spined loach population from Yakkan-R. had small erythrocytes [51]. The population belonged to the Y94 race and exhibited lower DNA content than the Y94 population from Saba-R.as confirmed in this study. To understand the factors that contribute to a reduction in DNA content in detail, additional analysis of populations within the chromosome race is required.

Although mtDNA analyses that can only trace maternal lineages have limitations in uncovering genome composition and chromosomal dynamics in allopolyploids, its independent inheritance from the nuclear genome could provide some insights into rapid rediploidization processes and distribution pattern formation of each chromosomal race in *Yamato* spined loach. Further studies using nuclear markers and molecular cytogenetic approaches, such as comparative genomic hybridization, can help reveal the detailed processes of rediploidization.

## Conclusions

This study demonstrates an ongoing, parallel process of chromosomal reorganization following polyploidization in the *Yamato* spined loach. Mitochondrial phylogenetic analyses revealed shallow differentiation and the retention of ancestral polymorphism across races, consistent with rapid rediploidization. Races with fewer chromosomes were positioned in derived clusters, suggesting a gradual reduction in chromosome number during the evolution of the *Yamato* spined loach. Geographically separated populations sharing the same chromosome number showed divergent mitochondrial haplotypes and differed in karyotype structure, providing evidence for parallel rediploidization in independently evolving lineages of the *Yamato* spined loach. Flow cytometry revealed no positive correlation between chromosome number and relative DNA content, suggesting that karyotype rearrangements, rather than the loss of specific chromosomes, contributed to the reduction in chromosome number and fundamental number.

## Supporting information

S1 Table Sampling locations, chromosome numbers and karyotype of the individuals, and mitochondrial haplotypes of *Yamato* spined loach (*Cobitis* sp. ‘yamato’ species complex). Races are arranged in a counterclockwise sequence according to their geographic distribution, beginning with the northernmost locality.
(XLSX)

S2 Table The number of individuals for karyotype, flow cytometric, and mtDNA analysis. (XLSX)

S3 Table Accession numbers of haplotypes used in this study. (XLSX)
